# To what extent do dietary costs explain socio-economic differences in dietary behavior?

**DOI:** 10.1186/s12937-020-00608-x

**Published:** 2020-08-24

**Authors:** Jody C. Hoenink, Joline W. J. Beulens, Marjolein C. Harbers, Jolanda M. A. Boer, S. Coosje Dijkstra, Mary Nicolaou, Yvonne T. van der Schouw, Ivonne Sluijs, W. M. Monique Verschuren, Wilma Waterlander, Joreintje D. Mackenbach

**Affiliations:** 1 Department of Epidemiology and Data Science, Amsterdam Public Health Research Institute, Amsterdam UMC, Vrije Universiteit Amsterdam, De Boelelaan, 1117 Amsterdam, the Netherlands; 2 Julius Center for Health Sciences and Primary Care, University Medical Center Utrecht, Utrecht University, Utrecht, the Netherlands; 3 National Institute for Public Health and the Environment (RIVM), Bilthoven, the Netherlands; 4 Department of Health Sciences, Faculty of Science, Vrije Universiteit Amsterdam, Amsterdam Public Health research institute, Amsterdam, the Netherlands; 5 Department of Public and Occupational Health, Amsterdam Public Health Research Institute, Amsterdam UMC, University of Amsterdam, Meibergdreef 9, Amsterdam, the Netherlands

**Keywords:** Food prices, Mediation analysis, Socioeconomic inequalities, Dietary quality

## Abstract

**Background:**

Low socio-economic position is associated with consumption of lower quality diets, which may be partly explained by the cost of healthier diets. Therefore, we aimed to investigate the mediating role of dietary costs in the association between educational level and diet quality.

**Methods:**

We used cross-sectional data from Dutch older adults (*N* = 9399) in the EPIC-NL cohort. Participants provided information about their own and their partners’ highest attained educational level (as proxy for socio-economic position). Dietary behavior was assessed using a food frequency questionnaire from which we derived two diet-quality scores, including the Dutch Healthy Diet index 2015 (DHD15-index) and the Dietary Approaches to Stop Hypertension (DASH) diet. Dietary cost estimates were based on food price data from food stores, and linked to reported consumption of food items. Multiple regression analyses and bootstrapping were used examine the mediating role of dietary cost in the association between educational level and diet quality.

**Results:**

Mean age of participants was 70 (SD: 10) years and 77% were women. Dietary costs significantly mediated the association between educational level and diet quality, except for high versus middle individual educational level and the DHD15-index. Depending on the dietary and educational indicator, dietary costs explained between 2 and 7% of the association between educational level and diet quality. Furthermore, associations were found to be modified by sex and age. For the DHD15-index, mediation effects were only present in females and adults older than 65 years, and for the DASH diet mediation effects were only present in females and strongest amongst adults older than 65 years compared to adults younger than 65 years.

**Conclusion:**

Dietary costs seems to play a modest role in explaining educational differences in diet quality in an older Dutch population. Further research is needed to investigate which other factors may explain SEP differences in diet quality.

## Background

The role of diet in the development of non-communicable chronic diseases (NCDs) is well established [1, 2]. Individuals with a lower socio-economic position (SEP) have an increased risk of developing diet-related chronic diseases [3, 4], which is partly explained by socio-economic differences in diet [5]. Indeed, studies demonstrated that individuals with a low SEP on average have unhealthier dietary behaviors and adhere less often to dietary guidelines compared to individuals with a higher SEP [6, 7].

The cost of eating a healthy diet may play an important role in explaining socio-economic differences in diet quality [7–9]. Price is an important factor in food choice, which is especially the case for individuals with a lower SEP [10]. Similarly, for individuals with a lower SEP, the perceived cost of healthy foods such as fruit, vegetables and fish is an important barrier for meeting dietary guidelines [11, 12]. In addition, a meta-analysis showed that, on average, healthier dietary patterns were more costly than less healthy dietary patterns [13]. This suggests that both perceived and actual food prices, potentially in combination with limited individual food budgets, may indeed constrain individuals with a lower SEP in consuming a high-quality diet.

A large body of literature has investigated the relationship between SEP and dietary behaviors, while few have studied the mechanisms by which SEP influences dietary outcomes. Gaining a better understanding of the explanatory role of dietary behaviors in the association between educational level and diet quality would help to establish the potential contribution of price interventions and policies on socio-economic differences in diet and consequently health. There is already limited evidence from two United States (US) and one European study showing that dietary costs partly explain socio-economic differences in diet quality [14–16]. However the study conducted in England used purchasing data instead of dietary intake data and it is unknown whether the results from the US studies prevail in European countries such as the Netherlands. Food price levels and socio-economic differences in health may differ between the US and Europe [17]. Even within European countries, the magnitude of inequalities in health associated with SEP differ [3]. Therefore, the current study aimed to investigate the mediating role of dietary costs in the association between educational level and diet quality in an older Dutch population.

## Methods

### Study population

**Fig. 1:**
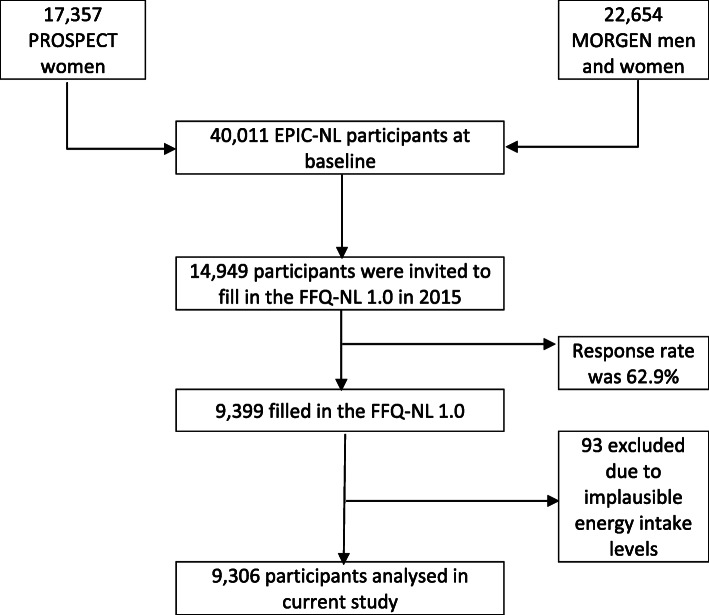
Flowchart of the study population

The flowchart of the study population is visualized in Fig. 1. This cross-sectional study used data from the European Prospective Investigation into Cancer and Nutrition cohort in the Netherlands (EPIC-NL) [18]. EPIC-NL consists of two Dutch cohorts, namely the Prospect cohort and MORGEN cohort. Participants from the Prospect cohort were females aged 50–70 years recruited from the national breast cancer screening program in Utrecht and its surroundings, while participants from the MORGEN cohort were aged 20–59 years, and selected from random samples of the Dutch population in three large cities in the Netherlands [18]. At baseline (1993–1997), participants filled in questionnaires and underwent a physical examination. The participants have been followed since for changes in risk factors and occurrence of chronic diseases.

In 2015, respondents to the 2011 questionnaire on electromagnetic radiation (EMR) [18] who were still alive, living in the Netherlands and who gave informed consent to be re-contacted (*n* = 13,421) were invited to fill out a Food Frequency Questionnaire (FFQ). Participants from the Doetinchem cohort did not receive the EMR questionnaire. Therefore, participants who at that time already participated in the 6th round of the Doetinchem cohort study (*n* = 1528) were invited to fill in the FFQ. The response rate to the FFQ from 2015 was 62.9% (*n* = 9399). For the current study, participants were excluded if they had implausible energy intake levels according to the lower or upper 0.5% ratio of reported energy intake over estimated energy requirement (*n* = 93) [19]. Ultimately, data from a total of 9306 participants was used for analysis in the current study.

### Dietary assessment

Dietary intake was assessed in 2015 using the validated FFQ-NL 1.0 [20]. The FFQ-NL 1.0 contained questions regarding the consumption and preparation methods of 160 food items during the prior year. Intake for each food item was reported in frequencies ranging from “never” to “7 days a week”. The servings were specified in terms of household measures or units of common portions such as one egg and one slice of bread. Using the 2011 computerized Dutch food composition table, dietary intake data yielded dietary energy intake (MJ and kcal) and the average daily intake of 43 macro- and micronutrients [20]. This FFQ was validated against on average 2.7 (range 1–5) telephone-based 24 h recalls as well as biomarkers in 24-h urine and blood samples. Correlation coefficients based on estimates of the FFQ and 24 h recalls ranged between 0.18 for fats, oils and sauces and 0.99 for soy and vegetarian products [20].

### Adherence to dietary guideline measures

Using the dietary intake data, adherence to the Dutch Dietary Guidelines was assessed with the Healthy Diet index 2015 (DHD15-index). The DHD15-index is developed for the Dutch population and is based on absolute intakes of selected foods. To allow comparison with international literature, adherence to the Dietary Approaches to Stop Hypertension (DASH) diet was also assessed, and represents a relative measure of dietary quality [21].

The DHD15-index was calculated as described by Looman et al. [22]. Briefly, The Health Council of the Netherlands released updated Dutch Food-based dietary guidelines in 2015, in which all evidence on nutrients, foods, and dietary patterns in relation to the most important chronic diseases and related risk-factors was integrated. To measure adherence to these guidelines, the DHD15-index was constructed. The index consists of 15 components (vegetables, fruits, whole grains, legumes, nuts, dairy, fish, tea, fats and oils, coffee, red meat, processed meat, sweetened beverages and fruit juices, alcohol and salt) which were assigned a score based on absolute intake of the specific food group. For all components a minimum of 0 points and a maximum of 10 points could be allocated, resulting in a total score ranging from 0 to 150 points, with higher score indicating better adherence to the guidelines. In EPIC-NL, data regarding filtered coffee was not available and estimates of salt intake were not included given the complexity of measuring intake using FFQs. Also, as previous studies excluded alcohol beverages because of their disproportionate influence on dietary costs due to their high price [23, 24], this component was excluded from the DHD15-index in the main analyses, thus the DHD15-index ranged from 0 to 120. Supplementary Table 1 includes the components of the DHD15-index and their minimum and maximum score values.

The DASH diet was originally developed as an intervention to prevent hypertension [25]. The DASH diet focuses on 8 components: high intake of fruits, vegetables, nuts and legumes, low-fat dairy products and whole grains, and low intake of sodium, sugary sweetened beverages, and red and processed meats [21]. The components of the DASH diet as proposed by Fung et al. were used to calculate adherence to the DASH diet [21]. For each component, participants were classified into quintiles according to their intake ranking. For components where a high intake is desired, participants in quintile 1 received one point and participants in quintile 5 received five points. For components where a low intake is desired, scoring was reversed (i.e. quintile 1 equals 5 points and quintile 5 equals 1 point). Participants in the quintiles between the lowest and highest quintiles received a score of 2, 3 or 4. Then, component scores were summed to obtain an overall DASH adherence score for each individual participant ranging from 7 to 35 (as previously mentioned, the sodium component was not calculated in this study). A higher score indicated better adherence to the DASH diet. Supplementary Table 2 includes the components of the DASH diet and the mean intake for the lowest and highest quintiles.

### Dietary cost measure

Individual dietary costs were derived using established methods [8, 14, 15]. A Dutch food price database was used to link food prices to individual food consumption as measured with the FFQ. A detailed description of the construction of this food price database can be found elsewhere [26]. Briefly, the food price database was developed in the summer of 2017 and covered 902 prices of foods based on a commonly used FFQ in Dutch cohorts [27]. This database included the retail prices of the lowest, non-promotional prices collected from two Dutch supermarket chains (i.e., a high segment supermarket Albert Heijn and a discount supermarket Lidl) in Amsterdam, the Netherlands. All prices in the food price database were adjusted for preparation and waste and were expressed in Euros (€) per 100 g edible portion [28]. Approximately 47% of all food products (*n* = 649) underlying the FFQ-NL 1.0 could directly be linked to the retail prices in our database. For 581 products, we linked the price of a comparable product to the consumed foods. For example, we used the price for Roosvicee syrup for both regular and multivitamine Roosvicee syrup. A few products (*n* = 78) that were assessed during the dietary intake assessment in 2015 were no longer sold when collecting the retail food prices in 2017. Furthermore, for a small number of foods (*n* = 27), no comparable product with a known price could be found in the database. Thus, the lowest available, non-promotional prices for these products were newly collected from two supermarket chains holding the largest market shares in the Netherlands (i.e. Albert Heijn and Jumbo). Once the dataset was complete, dietary costs were calculated by combining data from the food price database with the FFQ-NL 1.0 intake data. Each composite food item in the FFQ (total of 160 items) consists of a number of individual food items, weighted according to their relative contribution to the intake of that food in the Netherlands. We used the same weighting factor to calculate the cost of each composite FFQ food item. The variable obtained for each participant was the average monetary value of their habitual diet (reported dietary intake in the last year) in euros per day. Additional information regarding the food price database and the linking to FFQ data can be found in Supplementary file 1.

### Educational level measures

Based on the highest level of completed school education reported at baseline (1993–1997), three educational groups were constructed (low, middle and high) for participants and (if applicable) their partner. Those who completed primary education or intermediate vocational education were classified as having a low educational level, those who completed higher secondary education were classified as having a middle educational level and those who completed higher vocational education or university were classified as having a high educational level. Because educational level may vary for different birth cohorts and given the high proportion of older women whose own educational level may not reflect their SEP accurately [29], their partner’s educational level was used in addition to construct a household education level variable as a second indicator of SEP. Thus, two educational level variables were used for analyses: highest obtained individual educational level and highest household educational level (the highest attained educational level for either the participant or the partner of the participant). If a participant did not have a partner, then their own educational level was used as the highest attained educational level for the household.

### Covariates

Self-reported data on age and sex were obtained using the baseline questionnaire. Additionally, energy intake was derived from the dietary intake data obtained from the FFQ-NL 1.0. Study recruitment area (Amsterdam, Doetinchem, Utrecht and Maastricht) was also used as covariate. Because the association between SEP and diet quality differed for males versus females and adults from varying age groups in earlier studies, effect modification by age and sex was investigated [12, 30].

### Statistical analyses

**Fig. 2:**
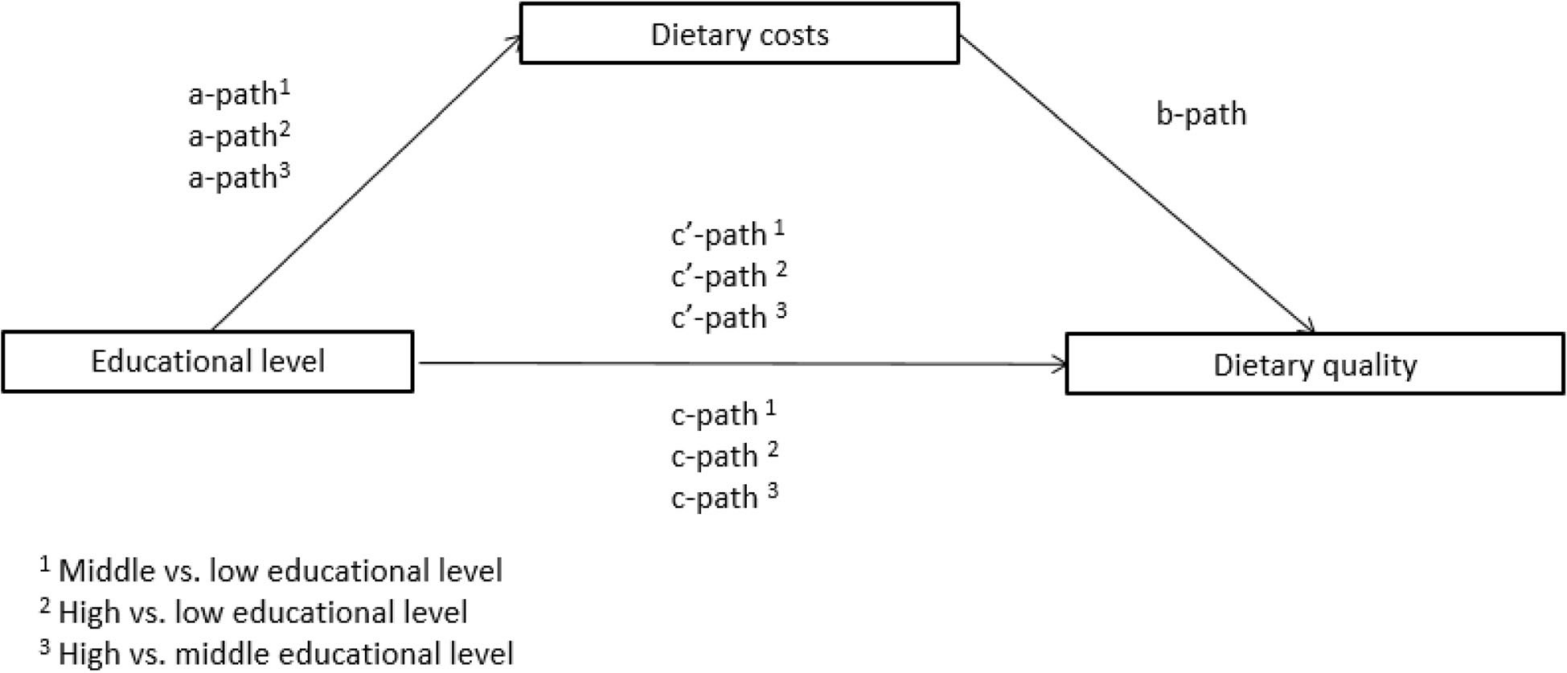
Pathways through which dietary cost may mediate the association between educational level and dietary quality. The c-paths are the associations between individual and household educational level and the dietary quality measures separately. The a-path is the association between the educational level variables and the potential mediator dietary cost. The b-paths and c’-paths are the associations between dietary cost and the dietary quality measures adjusted for the educational level variables (b-path) and between the educational level measures and the dietary quality measures adjusted for dietary cost (c’-path)

Descriptive statistics were computed for all variables, using percentages and means with standard deviations. Item-nonresponse ranged from 0% (age, sex, study center, DHD15-index and DASH score) to 0.33% (individual educational level). Complete case analysis was used for all analyses. Using the Hayes SPSS-macro PROCESS [31], the mediating role of dietary cost in the association between individual and household educational level and adherence to the diet quality measures (i.e. the DHD15-index and DASH diet) were assessed using multiple linear regression analyses. Figure 2 illustrates the mediating role of dietary cost in the association between individual and household educational level and diet quality. Instead of running all regression analyses separately, the package PROCESS allowed for an easier and faster estimation of the paths by running these simultaneously. Total effects were estimated by assessing the associations between individual and household educational level and the diet quality measures separately (c-path). Also, the association between the educational level variables and the potential mediator dietary cost was assessed (a-path). Furthermore, the associations between dietary cost and the diet quality measures adjusted for the educational level variables (b-path) and between the educational level measures and the diet quality measures adjusted for dietary cost (c’-path) were assessed. All analyses were adjusted for the covariates age, sex, study center and energy intake. Modification by age and sex was assessed using interaction terms age and both educational level measures and sex and both educational level measures on the c-path, a-path, b-path and c’-path. If one of the interaction terms was significant in at least one of the paths, associations were stratified by that effect modifier. In order to assess whether dietary cost indeed mediated the association between individual and household educational levels and the DHD15-index and the DASH score, the indirect effect and the corresponding 95% confidence interval were assessed. The indirect effects were calculated by multiplying the regression coefficient of the a-path with the regression coefficient of the b-path, which is equal to subtracting the regression coefficient of the c’-path from the regression coefficient of the c-path. The bootstrapped 95% confidence intervals around the indirect effects were based on 5000 bootstrap resamples. Statistical significance of the indirect effects was defined by the inclusion or exclusion of the value zero in the upper and lower bound of the bias corrected 95% bootstrap confidence intervals. As recommended by Hayes [31], the proportion mediated (a*b divided by a*b + c’) was calculated, but only if *1)* significant mediation was found, *2)* the total (c path) and indirect (a-path * b-path) effect had the same direction and *3)* if the indirect effect was smaller than the total effect. Sensitivity analysis was conducted by excluding participants (*n* = 529 for individual educational level and *n* = 531 for household educational level) below the age of 30 at the time of the baseline questionnaire (i.e. between 1993 and 1997), since their educational attainment may have changed between baseline and follow-up. In addition, sensitivity analyses were conducted including the alcohol component of the DHD15-index. All analyses were conducted with SPSS 25.0, and an α-level of 0.05 was used to test for statistical significance.

## Results

Participant characteristics by individual and household educational level are presented in Table 1. Mean age of all participants was 69.6 (SD 10.0) years old and the majority of participants were female (77.4%). Regarding individual educational level, 58.6% had a low educational level, 10.8% had a medium educational level and 30.6% had a high educational level. When taking the household educational level measure, the percentage of participants that was categorized as highly educated increased to 42.6%, whilst the percentage of participants that was categorized as low educated decreased to 46.5%. Mean energy intake was lowest for participants with a low individual and household educational level. Mean dietary cost (€ per day), the DHD15-index and the DASH score increased with increasing individual and household educational levels.

**Table 1:** Sociodemographic characteristics by individual and household educational level of the EPIC-NL study population

Sociodemographic characteristics	Low education level (*N* = 5435)	Middle education level (*N* = 1002)	High education level (*N* = 2838)	Low household education level (*N* = 4316)	Middle household education level (*N* = 1012)	High household education level (*N* = 3954)	Total (*N* = 9306)
Mean age in years (SD)	71.6 (8.8)	64.6 (13.2)	67.3 (9.9)	71.6 (8.8)	65.1 (13.1)	68.4 (9.9)	69.6 (10.0)
Sex (% female)	47.3	8.3	21.8	36.7	7.8	32.8	77.4
Mean energy intake in kcal/day (SD)	1850 (652)	1999 (646)	2023 (630)	1847 (662)	2017 (693)	1973 (616)	1919 (650)
Study center (in %)							
Amsterdam	6.6	2.9	8.7	5.6	2.6	10.0	18.1
Doetinchem	7.2	1.0	2.4	6.2	1.2	3.2	10.5
Maastricht	11.4	2.9	6.8	9.4	3.0	8.7	21.1
Utrecht	33.4	4.2	12.7	25.4	4.2	20.7	50.3
Mean dietary cost in €/day (SD)	4.9 (1.7)	5.4 (1.6)	5.5 (1.6)	4.8 (1.7)	5.3 (1.7)	5.4 (1.6)	5.1 (1.7)
DHD15-index (SD)^a^	63.2 (15.5)	67.2 (15.9)	72.0 (15.2)	62.2 (15.5)	65.3 (15.7)	70.9 (15.4)	66.2 (16.0)
DASH score (SD)^b^	20.1 (4.3)	21.2 (4.3)	22.5 (4.2)	20.0 (4.3)	20.8 (4.3)	22.1 (4.3)	21.0 (4.4)

*Abbreviations*: *SD* Standard deviation

^a^DHD15 index without the alcohol component ranging from 11.43 to 109.78

^b^DASH score ranging from 8 to 35

### Effects of educational level on dietary cost (a-path) and dietary cost on dietary quality (b-path)

Mostly, higher individual and household educational levels were statistically significantly associated with higher dietary costs (Supplementary Table 3; a-path). In turn, higher dietary cost was statistically significantly associated with higher DHD15-index and DASH scores (Supplementary Table 3; b-path). For individual educational level, a €1 increase in daily dietary costs was associated with a 0.74-point higher DHD15-index and a 0.29-point higher DASH score. These results are similar for household educational levels for both diet quality measures.

### Effect of educational level on dietary quality (c-path)

Higher individual and household educational levels were statistically significantly associated with higher DHD15-index and DASH scores (Table 2; total effect). Participants with an individual middle educational level scored 3.96 (95%CI 2.93; 5.00) and participants with a high educational level scored 9.07 (95%CI 8.37; 9.77) points higher on the DHD15-index compared to participants with a lower educational level. Participants with an individual high educational level scored 5.10 (95%CI 4.01; 6.19) points higher on the DHD15-index compared to participants with a middle educational level. For the DASH diet, participants with an individual middle educational level scored 1.00 (95%CI 0.71; 1.29) and participants with an individual high educational level scored 2.25 (95%CI 2.06; 2.45) points higher on the DASH diet compared to participants with a low educational level. Participants with an individual high educational level scored 1.25 (95%CI 095; 1.56) points higher on the DASH diet compared to participants with a middle educational level. These results were similar for household educational level for both diet quality measures.

**Table 2:** Results regarding the mediating role of dietary cost in the association between individual and household educational level and the DHD15-index and DASH score

Independent variable	Mediator	Dependent variable	Total effect (c-path)		Direct effect (c’-path)		Indirect effect		Proportion mediated
			β	95%CI	β	95%CI	β	Bootstrap 95%CI	
	Dietary cost (€/d)	DHD15- index (score)	Individual educational level						
Middle vs. low			3.96*	2.93; 5.00	3.78*	2.75; 4.82	0.18*	0.10; 0.28	4.5
High vs. low			9.07*	8.37; 9.77	8.86*	8.16; 9.57	0.20*	0.11; 0.30	2.2
High vs. middle			5.10*	4.01; 6.19	5.07*	4.00; 6.16	0.02	−0.03; 0.09	N/A
			Household educational level						
Middle vs. low			2.96*	1.91; 4.01	2.84*	1.79; 3.89	0.12*	0.05; 0.21	4.1
High vs. low			8.25*	7.59; 8.90	8.04*	7.37; 8.70	0.21*	0.11; 0.32	2.5
High vs. middle			5.20*	4.15; 6.24	5.28*	4.24; 6.33	0.09*	0.03; 0.16	1.7
		DASH diet (score)	Individual educational level						
Middle vs. low			1.00*	0.71; 1.29	0.93*	0.64; 1.22	0.07*	0.04; 0.10	7.0
High vs. low			2.25*	2.06; 2.45	2.17*	1.98; 2.37	0.08*	0.05; 0.11	3.6
High vs. middle			1.25*	0.95; 1.56	1.24*	0.94; 1.54	0.01	−0.01; 0.03	N/A
			Household educational level						
Middle vs. low			0.75*	0.46; 1.05	0.71*	0.41; 1.00	0.05*	0.03; 0.08	6.6
High vs. low			2.02*	1.84; 2.21	1.94*	1.76; 2.12	0.08*	0.05; 0.11	4.0
High vs. middle			1.27*	0.98; 1.56	1.23*	0.94; 1.52	0.03*	0.01; 0.06	2.4

*Abbreviations*: *B* beta regression coefficient, *CI* confidence interval, *N/A* Not Applicable

^a^Sample size for analyses with individual educational level is 9275

^b^Sample size for analyses with household educational level is 9282

*P < 0•05

All analyses were adjusted for age, sex, study center and energy intake

Proportion mediated was not calculated for non-significant indirect effects

### Mediating role of dietary cost

The association between individual and household educational level and both diet quality measures persisted after controlling for the mediator dietary cost (Table 2; direct effect). For example, household middle and high educational levels were associated with a 0.93 (95%CI 0.64; 1.22) and 2.17 (95%CI 1.98; 2.37) higher DASH score compared to household low educational level, respectively. The difference between individual middle and high educational level on the DASH diet after adjusting for dietary cost was 1.24 (95%CI 0.94; 1.54).

Regarding the pathway towards the DHD15-index (Table 2; indirect effect), dietary cost statistically significantly mediated the association between middle and high individual educational levels and the DHD15-index. These effects accounted for 4.5 and 2.2% of the difference in the DHD15-index score between middle and high versus low individual educational levels, respectively (Supplementary Table 3; proportion mediated). No mediating role of dietary cost in the association between high versus middle individual educational level and the DHD15-index was found. For household educational level, the association between middle and high household educational levels with dietary cost statistically significantly mediated the association with the DHD15-index (Table 2; indirect effect). These effects accounted for between 1.7 and 4.1% of the difference in the DHD15-index score depending on the educational level, with the lowest proportion mediated found when comparing high to middle household educational levels (Table 2; proportion mediated). Overall, the results regarding the associations between both individual and household educational level with the DASH score as the diet quality indicator were comparable to the results found with the DHD15-index.

### Modification by age and sex

Both sex and age were identified as modifiers in the educational level – dietary cost – diet quality pathway. Tables 3 and 4 include the results regarding the indirect effects by strata of age and sex. Full results of the mediation analyses separate for participants younger than 65 years and older than 65 years, and for males and females, can be found in the Supplementary Tables 4 through 7, respectively.

**Table 3:** Results regarding the mediating role of dietary cost in the association between individual and household educational level and the DHD15-index and DASH score for participants within varying age groups

Independent variable	Mediator	Dependent variable	≤ 65 years^**a**^			> 65 years^**b**^		
			Indirect effect		Proportion mediated	Indirect effect		Proportion mediated
			β	Bootstrap 95%CI		β	Bootstrap 95%CI	
	Dietary cost (€/d)	DHD15- index (score)	Individual educational level					
Middle vs. low			0.07	−0.03; 0.20	N/A	0.25*	0.13; 0.40	6.9
High vs. low			0.07	−0.03; 0.20	N/A	0.27*	0.15; 0.41	3.3
High vs. Middle			0.01	−0.05; 0.07	N/A	0.02	−0.07; 0.11	N/A
			Household educational level					
Middle vs. low			0.04	−0.04; 0.15	N/A	0.18*	0.08; 0.30	6.3
High vs. low			0.07	−0.07; 0.22	N/A	0.27*	0.15; 0.40	3.7
High vs. Middle			0.03	−0.03; 0.13	N/A	0.09*	0.02; 0.19	2.1
		DASH diet (score)	Individual educational level					
Middle vs. low			0.04*	0.01; 0.08	3.2	0.09*	0.06; 0.14	10.5
High vs. low			0.04*	0.01; 0.08	1.5	0.10*	0.07; 0.14	5.0
High vs. Middle			0.00	−0.03; 0.03	N/A	0.01	−0.02; 0.04	N/A
			Household educational level					
Middle vs. low			0.02	−0.00; 0.06	N/A	0.07*	0.03; 0.10	10.0
High vs. low			0.05*	0.01; 0.09	1.9	0.10*	0.06; 0.14	5.8
High vs. Middle			0.02	−0.00; 0.06	N/A	0.03*	0.01; 0.07	2.9

*Abbreviations*: *B* beta regression, *CI* confidence interval, *N/A* Not Applicable

^a^*N* = 2413 and *N* = 2418 for individual and household educational level, respectively

^b^*N* = 6862 and *N* = 6864 for individual and household educational level, respectively

* *P* < 0•05

All analyses were adjusted for energy intake, study center and sex.

Proportion mediated was not calculated for non-significant indirect effects.

**Table 4:** Results regarding the mediating role of dietary cost in the association between individual educational and household educational level and the DHD15-index and DASH score for females and males separately

Independent variable	Mediator	Dependent variable	Females^**a**^			Males^**b**^		
			Indirect effect		Proportion mediated	Indirect effect		Proportion mediated
			β	Bootstrap 95%CI		β	Bootstrap 95%CI	
	Dietary cost (€/d)	DHD15- index (score)	Individual educational level					
Middle vs. low			0.27*	0.15; 0.40	6.8	−0.02	− 0.18; 0.10	N/A
High vs. low			0.28*	0.18; 0.40	3.3	−0.03	− 0.21; 0.14	N/A
High vs. Middle			0.01	−0.07; 0.10	N/A	−0.01	−0.08; 0.06	N/A
			Household educational level					
Middle vs. low			0.19*	0.10; 0.31	7.1	−0.02	−0.14; 0.08	N/A
High vs. low			0.29*	0.18; 0.42	3.8	−0.04	−0.24; 0.15	N/A
High vs. Middle			0.10*	0.02; 0.19	2.0	−0.02	−0.14; 0.09	N/A
		DASH diet (score)	Individual educational level					
Middle vs. low			0.10*	0.06; 0.15	11.0	0.00	−0.04; 0.04	N/A
High vs. low			0.11*	0.07; 0.14	5.4	0.00	−0.05; 0.05	N/A
High vs. Middle			0.01	−0.03; 0.04	N/A	0.00	−0.02; 0.02	N/A
			Household educational level					
Middle vs. low			0.08*	0.04; 0.11	11.7	0.00	−0.03; 0.03	N/A
High vs. low			0.11*	0.08; 0.15	5.8	0.00	−0.05; 0.05	N/A
High vs. Middle			0.04*	0.01; 0.07	3.4	0.00	−0.03; 0.04	N/A

*Abbreviations*: *B* beta regression coefficient, *CI* confidence intervals, *N/A* Not Applicable

^a^*N* = 7175 and *N* = 7182 for individual and household educational level, respectively

^b^*N* = 2100 for both individual and household educational level

*P < 0•05

All analyses were adjusted for age, study center and energy intake

Proportion mediated was not calculated for non-significant indirect effects

For the diet quality measure the DHD15-index, sub-group analyses showed significant mediating effect by dietary cost in participants older than 65 years. For participants younger than 65 years, dietary cost did not play a statistically significant role in explaining educational differences in diet quality (Table 3). For the DASH diet, stronger mediating effects were found for participants older than 65 years compared to participants younger than 65 years (Table 3). Within both dietary quality measures, sub-group analyses showed that dietary cost explained between 3 and 12% of the differences between educational level and dietary quality for females, while no mediation effect was found for males (Table 4).

### Sensitivity analyses

Sensitivity analyses excluding participants below the age of 30 years at baseline show similar results compared to the main analyses with all participants (Supplementary Table 8). Dietary cost accounted for 2 to 8% of the individual and household educational level differences in diet quality scores. This is similar to the results found in the entire sample, where the proportion mediated ranged from 2 to 7%.

The results for the DHD15-index including the alcohol component can be found in Supplementary Table 9. Contrary to the results without the alcohol component, dietary cost was negatively associated with the DHD15-index including the alcohol component. For example, for individuals with a middle compared to lower educational level, a €1 increase in daily dietary costs was associated with a 0.78-point lower DHD15 score. Contrary to the results excluding the alcohol component from the DHD15-index, a statistically significant negative mediating effect of dietary cost in the association between educational level and the DHD15-index was found.

## Discussion

This study used a large cohort of older Dutch adults to investigate to what extent dietary costs explain educational differences in diet quality. We observed a modest association of individual and household educational level with diet quality as measured by adherence to the 2015 Dutch nutrition guidelines and adherence to the DASH diet. A small part of this association was explained by dietary costs, such that mediation effects were mostly only present in females and adults older than 65 years compared to males and adults younger than 65 years.

In line with previous findings [6, 30, 32], we observed that higher educational levels were associated with healthier dietary behaviors. We hypothesized that educational differences in diet quality would be partly explained by dietary costs, because high quality diets (e.g., diets containing plenty of fruits and vegetables and less red meats) tend to be more costly than lower quality diets [13, 33]. Indeed, a €1 increase in daily dietary costs was associated with a 0.29-point higher DASH score, which is comparable to the results of a previous study [34]. Dietary cost explained between 2 and 7% of the association between educational level and diet quality measures. The somewhat larger explanatory role of dietary cost in the association between educational level and diet quality for females (proportion mediated between 2 and 12%) and adults older than 65 (proportion mediated between 3 and 11%) may be explained by differences in competing factors such as perceived quality, price, taste, habits, intentions to eat healthily and family preferences for females versus males and older adults versus younger adults [35]. For example, a study among European adults showed that while price was among the top four important factors influencing food choices for females, this was not the case for males [35]. As noted in two previous studies [23, 24], the in- or exclusion of the alcohol component in the DHD15-index had a significant impact on the association between dietary costs and diet quality.

Compared to previous studies [14–16], this study found relatively small mediation effects by dietary cost in the association between educational level and diet quality measures for which the explanation may be two-fold. Firstly, the type of socio-economic indicator may play a role. Socio-economic indicators including income, educational level and occupation are not interchangeable, as they assess different aspects of SEP [36]. Educational level is thought to better reflect knowledge-related assets, while income may reflect material circumstances such as grocery shopping budget better [37]. While income and education were not differentially associated with daily dietary costs in a population from the United Kingdom [8], this may differ from country to country. A study by Aggarwal et al. found larger mediation effects with the SEP indicator income, e.g., 76% of the association between income and energy density and 36% of the association between income and the mean adequacy ratio (i.e., a truncated index of the percent of daily recommended intakes for key nutrients) could be explained by dietary cost [14]. In contrast, a study by Waterlander et al. found that level of income was not associated with food expenses [38]. This leads us to the second explanation, namely that in the Netherlands, dietary costs may play a smaller role in the association between SEP and dietary quality due to the relatively low cost of food compared to other countries and other consumer goods. According to European Union (EU), the prices of food and non-alcohol beverages in the Netherlands is around the EU average, which is lower compared to other West-European countries [39].

The limited explanatory power of dietary cost also warrants discussion about other potential factors that explain SEP inequalities in diet quality. Socio-economic inequalities are traditionally explained through three mechanisms; the material explanation, the behavioral explanation and the psychosocial explanation [40]. While several studies have examined these explanatory mechanisms separately, it would be of interest to study the relative contribution of dietary costs compared to other explanatory factors [41]. Other explanatory factors may include access to healthy foods, other aspects of the food environment and psychosocial resources such as knowledge of healthy eating, cooking skills, food literacy or social support. Thus, before developing new interventions, future studies should focus on providing insights into the most relevant causes and mechanisms that underlie socioeconomic differences in diet quality. It is only then that interventions can be adequately designed and tailored to the needs and capacities of the target population. Some interventions to date that have increased dietary quality without increasing socio-economic inequalities include subsidies on healthy foods [42] and sugar sweetened beverage taxes [43].

The results of this study should be interpreted within the context of its strengths and limitations. A first strength is the use of a detailed food price database that could be linked to individual food intake using established techniques. Second, we used a diet quality indicator that was relevant for the study population (DHD15) and a diet quality indicator that is widely used in the international literature (DASH). The first limitation is the use of data from an older Dutch population, limiting the generalizability of the results. However, given that data was collected across different regions in the Netherlands, the generalizability of the results can be increased to the entire older Dutch population. Second, mediation analyses were based on cross sectional data, and therefore we cannot make inferences about causality [14]. For example, it is possible that higher educational level leads to more dietary knowledge, in turn leading to a healthier diet, and that this healthier diet leads to higher dietary costs, and vice versa [16]. Third, the dietary cost measure (as derived from reported dietary intake with a fixed food price database) is not suitable for estimating actual expenditure, since price data regarding the cheapest available product, instead of the actual consumed product, was collected. However, our current measure of dietary costs is found to be moderately associated with actual food expenditure, and able to rank individuals on the basis of the monetary value of their diets [44]. Furthermore, our conservative approach of only using the lowest available prices is more likely to have led to an underestimation than to an overestimation of the mediating role of dietary cost in the educational level and diet quality pathway [16]. Fourth, the dietary cost measure data was collected 2 years after the dietary intake data. Within these 2 years, the prices of foods may have increased due to inflation. However, given that this type of time-varying bias would not systematically misclassify people of different dietary or social characteristics, the ranking of individuals has likely not changed in the period of 2015 to 2017. Fifth, while the validity of the FFQ used in this study has been documented, the limitations of FFQs are well-known [45]. One such limitation includes the fact that dietary intake may be under- or overreported. Underreporting may especially be the case for participants with a lower education [46]. However, what effect this may have on the results is currently unknown and beyond the scope of this paper. Sixth, unfortunately data on other important SEP proxies such as income was not collected, potentially resulting in an underestimation of the mediating role of dietary cost in the relation between SEP and dietary quality.

## Conclusion

Dietary costs seems to play a modest role in explaining educational differences in diet quality in an older Dutch population, such that mediation effects were mostly only present in females and adults older than 65 years compared to males and adults younger than 65 years. The results of this study suggest that further research is needed to investigate which other factors may explain SEP differences in diet quality. A multi-factorial approach is needed to tackle socio-economic differences in diet, not only focusing on the cost of food, but also other factors such as food knowledge and access to healthy foods.

## Supplementary information

**Additional file 1.** Methodology used to derive the lowest available food prices for participants of the HELIUS study.

**Additional file 2: Table S1.** DHD15-index components as used in the current study with the corresponding Dutch dietary guidelines and the minimum and maximum score values per component, adapted from Looman et al. **Table S2.** DASH diet components with the corresponding foods for each component and the mean intake for the lowest and highest quintiles, adapted from Fung et al. **Table S3.** Results regarding the mediating role of dietary cost in the association between individual and household educational level and the DHD15-index and DASH score. **Table S4.** Results regarding the mediating role of dietary cost in the association between individual and household educational level and the DHD15-index and DASH score for participants ≤65 years of age. **Table S5:** Results regarding the mediating role of dietary cost in the association between individual and household educational level and the DHD15-index and DASH score for participants over 65 years of age. **Table S6.** Results regarding the mediating role of dietary cost in the association between individual and household educational level and the DHD15-index and DASH score for females. **Table S7.** Results regarding the mediating role of dietary cost in the association between individual and household educational level and the DHD15-index and DASH score for males. **Table S8.** Results regarding the mediating role of dietary cost in the association between individual and household educational level and the DHD15-index and DASH score for participants older than 30 years at baseline. **Table S9.** Results regarding the mediating role of dietary cost in the association between individual and household educational level and the DHD15-index with alcohol component.

## Data Availability

The datasets used and/or analysed during the current study are available from the Principal Investigator of EPIC-NL (YTS) on reasonable request.

## Abbreviations

DASH: Dietary Approaches to Stop Hypertension
DHD15-index: Dutch Healthy Diet Index 2015
EMR: Electromagnetic Radiation
EPIC-NL: European Prospective Investigation into Cancer and Nutrition cohort in the Netherlands
FFQ: Food Frequency Questionnaire
NCDs: Non-communicable Chronic Diseases
US: United States
SEP: Socio-economic Position
SD: Standard Deviation
95%CI: 95% Confidence Interval

## References

[1] Forouzanfar M, Alexander LT, Anderson H, Bachman V, Biryukov S, Brauer M, Burnett RT, Casey DC, Coates M, Cohen A, Delwiche K (2015). Global, regional and national comparative risk assessment of 79 behavioural, environmental and occupational, and metabolic risks or clusters of risks in 188 countries, 1990-2013: a systematic analysis for the global burden of diseases study 2013. Lancet.

[2] Collaborators GBDD (2019). Health effects of dietary risks in 195 countries, 1990-2017: a systematic analysis for the global burden of disease study 2017. Lancet.

[3] Mackenbach JP, Stirbu I, Roskam AJR, Schaap MM, Menvielle G, Kunst AE (2008). Socioeconomic inequalities in health in 22 European countries. N Engl J Med.

[4] Marmot M (2005). Social determinants of health inequalitie. Lancet.

[5] Méjean C, Droomers M, van der Schouw YT, Sluijs I, Czernichow S, Grobbee DE, Beulens JW (2013). The contribution of diet and lifestyle to socioeconomic inequalities in cardiovascular morbidity and mortality. Int J Cardiol.

[6] Giskes K, Avendano M, Brug J, Kunst AE (2010). A systematic review of studies on socioeconomic inequalities in dietary intakes associated with weight gain and overweight/obesity conducted among European adults. Obes Rev.

[7] Dijkstra SC, Neter JE, Brouwer IA, Huisman M, Visser M (2014). Adherence to dietary guidelines for fruit, vegetables and fish among older Dutch adults; the role of education, income, and job prestige. J Nutr Health Aging.

[8] Mackenbach JD, Brage S, Forouhi NG, Griffin SJ, Wareham NJ, Monsivais P (2015). Does the importance of dietary costs for fruit and vegetable intake vary by socioeconomic position?. Br J Nutr.

[9] Darmon N, Drewnowski A (2015). Contribution of food prices and diet cost to socioeconomic disparities in diet quality and health: a systematic review and analysis. Nutr Rev.

[10] Steenhuis IH, Waterlander WE, de Mul A (2011). Consumer food choices: the role of price and pricing strategies. Public Health Nutr.

[11] Dijkstra SC, Neter JE, van Stralen MM, Knol DL, Brouwer IA, Huisman M, Visser M (2015). The role of perceived barriers in explaining socio-economic status differences in adherence to the fruit, vegetable and fish guidelines in older adults: a mediation study. Public Health Nutr.

[12] Beydoun MA, Wang Y (2008). How do socio-economic status, perceived economic barriers and nutritional benefits affect quality of dietary intake among US adults?. Eur J Clin Nutr.

[13] Rao M, Afshin A, Singh G, Mozaffarian D (2013). Do healthier foods and diet patterns cost more than less healthy options? A systematic review and meta-analysis. BMJ Open.

[14] Aggarwal A, Monsivais P, Cook AJ, Drewnowski A (2011). Does diet cost mediate the relation between socioeconomic position and diet quality?. Eur J Clin Nutr.

[15] Monsivais P, Aggarwal A, Drewnowski A (2012). Are socio-economic disparities in diet quality explained by diet cost?. J Epidemiol Community Health.

[16] Pechey R, Monsivais P (2016). Socioeconomic inequalities in the healthiness of food choices: exploring the contributions of food expenditures. Prev Med.

[17] Avendano M, Glymour MM, Banks J, Mackenbach JP (2009). Health disadvantage in US adults aged 50 to 74 years: a comparison of the health of rich and poor Americans with that of Europeans. Am J Public Health.

[18] Beulens JW, Monninkhof EM, Verschuren WM, van der Schouw YT, Smit J, Ocke MC, Jansen EH, van Dieren S, Grobbee DE, Peeters PH, Bueno-de-Mesquita HB (2010). Cohort profile: the EPIC-NL study. Int J Epidemiol.

[19] Fransen HP, Boer JMA, Beulens JWJ, de Wit GA, Bueno-de-Mesquita HB, Hoekstra J, May AM, Peeters PHM (2017). Associations between lifestyle factors and an unhealthy diet. Eur J Pub Health.

[20] Sluik D, Geelen A, de Vries JH, Eussen SJ, Brants HA, Meijboom S, van Dongen MC, Bueno-de-Mesquita HB, Wijckmans-Duysens NE, van 't van’t Veer P, et al: A national FFQ for the Netherlands (the FFQ-NL 1.0): validation of a comprehensive FFQ for adults. Br J Nutr 2016, 116**:**913–923. 10.1017/S0007114516002749 27452894

[21] Fung T, Chiuve S, McCullough M, Rexrode K, Logroscino G, Hu F (2008). Adherence to a DASH style diet and risk of coronary heart disease and stroke in women. Arch Intern Med.

[22] Looman M, Feskens EJ, de Rijk M, Meijboom S, Biesbroek S, Temme EH, de Vries J, Geelen A (2017). Development and evaluation of the Dutch healthy diet index 2015. Public Health Nutr.

[23] Timmins KA, Hulme C, Cade JE (2015). The monetary value of diets consumed by British adults: an exploration into sociodemographic differences in individual-level diet costs. Public Health Nutr.

[24] Pondor I, Gan WY, Appannah G. Higher dietary cost is associated with higher diet quality: a cross-sectional study among selected Malaysian adults. Nutrients. 2017;9. 10.3390/nu9091028 PMC5622788 28926947

[25] Appel LJ, Moore TJ, Obarzanek E, Vollmer WM, Svetkey LP, Sacks FM, Bray GA, Vogt TM, Cutler JA, Windhauser MM (1997). A clinical trial of the effects of dietary patterns on blood pressure. DASH collaborative research group. N Engl J Med.

[26] Mackenbach JD, Dijkstra SC, Beulens JWJ, Seidell JC, Snijder MB, Stronks K, Monsivais P, M N: Socio-economic and ethnic differences in the relation between dietary costs and dietary quality: the HELIUS study. Nutr J 2019, 18**:**21. 10.1186/s12937-019-0445-3 PMC6440156 30922320

[27] Beukers MH, Dekker LH, de Boer EJ, Perenboom CW, Meijboom S, Nicolaou M, de Vries JH, Brants HA (2015). Development of the HELIUS food frequency questionnaires: ethnic-specific questionnaires to assess the diet of a multiethnic population in the Netherlands. Eur J Clin Nutr.

[28] Donders Engelen MR, LJMvd H, KFAM H (1997). Maten gewichten en codenummers. Human Nutrition.

[29] Galobardes B, Shaw M, Lawlor DA, Lynch JW, Davey Smith G (2006). Indicators of socioeconomic position (part 1). J Epidemiol Community Health.

[30] Backholer K, Spencer E, Gearon E, Magliano DJ, McNaughton SA, Shaw JE, Peeters A (2016). The association between socio-economic position and diet quality in Australian adults. Public Health Nutr.

[31] Hayes A (2012). PROCESS: A versatile computational tool for observed variable mediation, moderation, and conditional process modeling.

[32] Schoufour JD, de Jonge EAL, Kiefte-de Jong JC, van Lenthe FJ, Hofman A, Nunn SPT, Franco OH (2018). Socio-economic indicators and diet quality in an older population. Maturitas.

[33] Ryden PJ, Hagfors L (2011). Diet cost, diet quality and socio-economic position: how are they related and what contributes to differences in diet costs?. Public Health Nutr.

[34] Monsivais P, Rehm C, Drewnowski A (2013). The DASH diet and diet costs among ethnic and racial groups in the United States. JAMA Intern Med.

[35] Lennernas M, Fjellstrom C, Becker W, Giachetti I, Schmitt A, de Winter AR, Kearney M (1997). Influences on food choice perceived to be important by nationally-representative samples of adults in the European Union. Eur J Clin Nutr.

[36] Braveman PA, Cubbin C, Egerter S, Chideya S, Marchi KS, Metzler M, Posner S (2005). Socioeconomic status in health research: one size does not fit all. JAMA.

[37] Galobardes B, Lynch J, Smith GD (2007). Measuring socioeconomic position in health research. Br Med Bull.

[38] Waterlander WE, de Haas WE, van Amstel I, Schuit AJ, Twisk JW, Visser M, Seidell JC, Steenhuis IH (2010). Energy density, energy costs and income - how are they related?. Public Health Nutr.

[39] Eurostat Statistics Explained. https://ec.europa.eu/eurostat/statistics-explained/index.php/Comparative_price_levels_of_consumer_goods_and_services. Accessed 19 Aug 2019.

[40] Townsend P, Phillimore P, Beattie A. Health and deprivation: inequality and the north: Routledge; 1988.

[41] Moor I, Spallek J, Richter M (2017). Explaining socioeconomic inequalities in self-rated health: a systematic review of the relative contribution of material, psychosocial and behavioural factors. J Epidemiol Community Health.

[42] Ni Mhurchu C, Blakely T, Jiang Y, Eyles HC, Rodgers A (2010). Effects of price discounts and tailored nutrition education on supermarket purchases: a randomized controlled trial. Am J Clin Nutr.

[43] Backholer K, Sarink D, Beauchamp A, Keating C, Loh V, Ball K, Martin J, Peeters A (2016). The impact of a tax on sugar-sweetened beverages according to socio-economic position: a systematic review of the evidence. Public Health Nutr.

[44] Rehm CD, Monsivais P, Drewnowski A (2011). The quality and monetary value of diets consumed by adults in the United States. Am J Clin Nutr.

[45] Drewnowski A (2001). Diet image: a new perspective on the food-frequency questionnaire. Nutr Rev.

[46] Macdiarmid J, Blundell J (1998). Assessing dietary intake: who, what and why of under-reporting. Nutr Res Rev.

