# Correction to: To what extent do dietary costs explain socio-economic differences in dietary behavior?

**DOI:** 10.1186/s12937-022-00767-z

**Published:** 2022-03-17

**Authors:** Jody C. Hoenink, Joline W. J. Beulens, Marjolein C. Harbers, Jolanda M. A. Boer, S. Coosje Dijkstra, Mary Nicolaou, Yvonne T. van der Schouw, Ivonne Sluijs, W. M. Monique Verschuren, Wilma Waterlander, Joreintje D. Mackenbach

**Affiliations:** 1 Amsterdam UMC, Vrije Universiteit Amsterdam, Department of Epidemiology and Biostatistics, Amsterdam Public Health Research Institute, De Boelelaan, 1117 Amsterdam, the Netherlands; 2 Julius Center for Health Sciences and Primary Care, University Medical Center Utrecht, Utrecht University, Utrecht, the Netherlands; 3 National Institute for Public Health and the Environment (RIVM), Bilthoven, the Netherlands; 4 Department of Health Sciences, Faculty of Science, Vrije Universiteit Amsterdam, Amsterdam Public Health Research Institute, Amsterdam, the Netherlands; 5 Amsterdam UMC, University of Amsterdam, Department of Public Health, Amsterdam Public Health Research Institute, Meibergdreef 9, Amsterdam, the Netherlands

**Correction to: Nutr J 19, 88 (2020)**

**https://doi.org/10.1186/s12937-020-00608-x**

Following publication of the original article [1], the authors reported an error in Table 1 where instead of the percentage females, the percentage males were reported. This has now been amended.

**Table 1:** Sociodemographic characteristics by individual and household educational level of the EPIC-NL study population

Sociodemographic characteristics	Low education level (***N***= 5,435)	Middle education level (***N***= 1,002)	High education level (***N***= 2,838)	Low household education level (***N***= 4,316)	Middle household education level (***N***= 1,012)	High household education level (***N***= 3,954)	Total (***N***= 9,306)
Mean age in years (SD)	71.6 (8.8)	64.6 (13.2)	67.3 (9.9)	71.6 (8.8)	65.1 (13.1)	68.4 (9.9)	69.6 (10.0)
Sex (% female)	52.7	91.7	78.2	63.3	92.2	67.2	77.4
Mean energy intake in kcal/day (SD)	1,850 (652)	1,999 (646)	2,023 (630)	1,847 (662)	2,017 (693)	1,973 (616)	1,919 (650)
Study center (in %)							
Amsterdam	6.6	2.9	8.7	5.6	2.6	10.0	18.1
Doetinchem	7.2	1.0	2.4	6.2	1.2	3.2	10.5
Maastricht	11.4	2.9	6.8	9.4	3.0	8.7	21.1
Utrecht	33.4	4.2	12.7	25.4	4.2	20.7	50.3
Mean dietary cost in €/day (SD)	4.9 (1.7)	5.4 (1.6)	5.5 (1.6)	4.8 (1.7)	5.3 (1.7)	5.4 (1.6)	5.1 (1.7)
DHD15-index (SD)^a^	63.2 (15.5)	67.2 (15.9)	72.0 (15.2)	62.2 (15.5)	65.3 (15.7)	70.9 (15.4)	66.2 (16.0)
DASH score (SD)^b^	20.1 (4.3)	21.2 (4.3)	22.5 (4.2)	20.0 (4.3)	20.8 (4.3)	22.1 (4.3)	21.0 (4.4)

*Abbreviations*: *SD* Standard deviation

^a^ DHD15 index without the alcohol component ranging from 11.43 to 109.78

^b^ DASH score ranging from 8 to 35

The original article [1] has been updated.
