# Supplementary material for: To what extent do dietary costs explain socio-economic differences in dietary behavior?
Source: Nutr J. 2020 Aug 24;19:88. doi: 10.1186/s12937-020-00608-x (PMC7446174; doi:10.1186/s12937-020-00608-x)
Supplement: Supplementary file 1 — Additional file 1. Methodology used to derive the lowest available food prices for participants of the HELIUS study. [file 12937_2020_608_MOESM1_ESM.pdf]

Additional File 1. Methodology used to derive the lowest available food prices for participants of the HELIUS study.

A more detailed description of this methodology is available in the methodology report. This can be downloaded from this website.

Briefly, food retailers that represented a wide range of available food products within Amsterdam, the Netherlands were identified by the Foodprice research team (JD Mackenbach, SC Dijkstra and M Nicolaou). The discount supermarket chain Lidl and the full-service supermarket chain Albert Heijn were used to collect most food price data. Also, food price data were collected from local (ethnic) shops such as a Turkish butcher and greengrocers. In total, prices were collected in 20 shops during July and August of 2017 (to prevent variation in food prices due to seasonality) and to be able to obtain the lowest price available in Amsterdam. Excel-sheets were used to collect the food price data. Items to be collected for the food price database were selected from four HELIUS Food Frequency Questionnaires (FFQs). Ultimately, the food price database consisted of 1,247 unique foods. Each individual food product underlying the HELIUS FFQ-items were translated to a specific food item in purchasable form. For example, 'potatoes without peel' was translated into 'potatoes'. Additionally, food items that essentially represented the same products were combined (e.g. different types of concentrated cordials). Ultimately, the prices for 902 products were collected for the food price database. Foods were sorted according to food group to facilitate speedy data collection. Data were collected on food price (€), unit (in KG or L), name of the product, date at which this price was collected and any remarks with regard to the product. For products in tin or glass jars, the net weight of the product as unit was used. When collecting the data, photos from each separate food items were collected if possible. If multiple options were available, the cheapest products were chosen. For packaged foods, the median package size was used. When items were on sale, the sale price was ignored and the everyday price was recorded. If a product was not available, a close match was used a substitute. After collecting food price data in the selected shops, data on food prices was combined into a single excel sheet with colours indicating the source of the price information. Prices per 100 grams were calculated based on the price and the unit of the product. The Dutch "Maten Gewichten en Codenummers 2003"(1) was used to calculate the edible portion per food. This report also included information on the weights of average non-packaged foods, such as zucchinis, and information on waste and gain/loss during preparation. The food prices of these 902 purchasable food items were then linked to the 1289 FFQ-NL 1.0 food items from the EPIC-NL study. Forty-seven percent of all food items (n= 649) underlying the FFQ-NL 1.0 could directly be linked to the retail prices in the food price database. A few products (n=78) that were assessed during the dietary intake assessment in 2015 were no longer available when collecting the retail food prices in 2017. Furthermore, for a small number of food items (n= 27), no comparable product with a known price could be found (e.g. kaki fruit, fennel and caviar) in the database. Thus, the lowest available, non-promotional prices for these products were newly collected from two supermarket chains holding the largest market shares in the Netherlands (i.e. Albert Heijn and Jumbo). For the remaining 581 food items, we linked the missing price to a comparable product within the database. For

example, the price of chocolate covered waffles was given the price for waffles from the food price database. A weighting system was used to calculate the price in €/100 gr edible portion for each weighted food item within the FFQ-NL 1.0. For example, the price for the food item pizza consisted of 80.7% ‘frozen pizza’ and 19.4% ‘mini pizzas’. Once the dataset was complete (i.e. when every item within the FFQ-NL 1.0 had a price), dietary costs were calculated by combining data from the food price database to the FFQ-NL 1.0 nutrient composition database. This nutrient composition database was used to calculate the nutrient intake of each individual based on their intake of each food product in the FFQ. The food price variable obtained from the steps above included the monetary value of each participants’ diet (reported dietary intake in the last week/month) in euros per day.

Reference list:

1. Donders-Engelen M, van der Heijden L, Hulshof K. Maten Gewichten en Codenummers 2003. Wageningen UR, Vakgroep Humane Voeding
