# Supplementary material for: To what extent do dietary costs explain socio-economic differences in dietary behavior?
Source: Nutr J. 2020 Aug 24;19:88. doi: 10.1186/s12937-020-00608-x (PMC7446174; doi:10.1186/s12937-020-00608-x)
Supplement: Supplementary file 2 — Additional file 2: Table S1. DHD15-index components as used in the current study with the corresponding Dutch dietary guidelines and the minimum and maximum score values per component, adapted from Looman et al. Table S2. DASH diet components with the corresponding foods for each component and the mean intake for the lowest and highest quintiles, adapted from Fung et al. Table S3. Results regarding the mediating role of dietary cost in the association between individual and household educational level and the DHD15-index and DASH score. Table S4. Results regarding the mediating role of dietary cost in the association between individual and household educational level and the DHD15-index and DASH score for participants ≤65 years of age. Table S5: Results regarding the mediating role of dietary cost in the association between individual and household educational level and the DHD15-index and DASH score for participants over 65 years of age. Table S6. Results regarding the mediating role of dietary cost in the association between individual and household educational level and the DHD15-index and DASH score for females. Table S7. Results regarding the mediating role of dietary cost in the association between individual and household educational level and the DHD15-index and DASH score for males. Table S8. Results regarding the mediating role of dietary cost in the association between individual and household educational level and the DHD15-index and DASH score for participants older than 30 years at baseline. Table S9. Results regarding the mediating role of dietary cost in the association between individual and household educational level and the DHD15-index with alcohol component. [file 12937_2020_608_MOESM2_ESM.docx]

**To what extent do dietary costs explain socio-economic differences in dietary behavior?**

Jody C. Hoenink^1^, Joline W.J. Beulens^1,2^, Marjolein C. Harbers^2^, Jolanda M.A. Boer^3^, S. Coosje Dijkstra^4^, Mary Nicolaou^5^, Yvonne T. van der Schouw^2^, Ivonne Sluijs^2^, W.M. Monique Verschuren^2,3^, Wilma Waterlander^1,5^, Joreintje D. Mackenbach^1^

^1^ Amsterdam UMC, Vrije Universiteit Amsterdam, Department of Epidemiology and Biostatistics, Amsterdam Public Health research institute, De Boelelaan 1117, Amsterdam, the Netherlands

^2^ Julius Center for Health Sciences and Primary Care, University Medical Center Utrecht, Utrecht University, Utrecht, the Netherlands

^3^ National Institute for Public Health and the Environment (RIVM), Bilthoven, the Netherlands

^4^ Department of Health Sciences, Faculty of Science, Vrije Universiteit Amsterdam, Amsterdam Public Health research institute, the Netherlands

^5^ Amsterdam UMC, University of Amsterdam, Department of Public Health, Amsterdam Public Health Research Institute, Meibergdreef 9, Amsterdam, the Netherlands

Table of contents

[Supplementary Table 1: DHD15-index components as used in the current study with the corresponding Dutch dietary guidelines and the minimum and maximum score values per component, adapted from Looman et al. 3](#_Toc41651449)

[Supplementary Table 2: DASH diet components with the corresponding foods for each component and the mean intake for the lowest and highest quintiles, adapted from Fung et al. 5](#_Toc41651450)

[Supplementary Table 3: Results regarding the mediating role of dietary cost in the association between individual and household educational level and the DHD15-index and DASH score 6](#_Toc41651451)

[Supplementary Table 4: Results regarding the mediating role of dietary cost in the association between individual and household educational level and the DHD15-index and DASH score for participants ≤ 65 years of age. 7](#_Toc41651452)

[Supplementary Table 5: Results regarding the mediating role of dietary cost in the association between individual and household educational level and the DHD15-index and DASH score for participants over 65 years of age. 8](#_Toc41651453)

[Supplementary Table 6: Results regarding the mediating role of dietary cost in the association between individual and household educational level and the DHD15-index and DASH score for females. 9](#_Toc41651454)

[Supplementary Table 7: Results regarding the mediating role of dietary cost in the association between individual and household educational level and the DHD15-index and DASH score for males. 10](#_Toc41651455)

[Supplementary Table 8: Results regarding the mediating role of dietary cost in the association between individual and household educational level and the DHD15-index and DASH score for participants older than 30 years at baseline 11](#_Toc41651456)

[Supplementary Table 9: Results regarding the mediating role of dietary cost in the association between individual and household educational level and the DHD15-index with alcohol component 13](#_Toc41651457)

# Supplementary Table 1: DHD15-index components as used in the current study with the corresponding Dutch dietary guidelines and the minimum and maximum score values per component, adapted from Looman et al.

| **Component** | **Food items in DHD15-index components** | **Dutch dietary guideline 2015** | **Minimum score of 0 points** | **Maximum score of 10 points** |
| --- | --- | --- | --- | --- |
| Vegetables | All vegetables except for potatoes and legumes | Consume at least 200 grams of vegetables everyday | 0 grams a day | ≥ 200 grams a day |
| Fruit | All fruits | Consume at least 200 grams of fruits everyday | 0 grams a day | ≥ 200 grams a day |
| Wholegrains^a^ | High-fibre bread | 1. Consume at least 90 grams of wholegrain products everyday | 0 grams a day | ≥ 90 grams a day |
|  |  | 2. Substitute refined cereal products by wholegrain products | Ratio of whole grains to refined grains ≤ 0.7 | Ratio of whole grains to refined grains ≥ 11 |
| Legumes | Beans and peas | Consume legumes every week | 0 grams a day | ≥ 10 grams a week |
| Nuts | Nuts, seeds and seeds | Consume at least 15 grams of unsalted nuts everyday | 0 grams a day | ≥ 15 grams a day |
| Dairy | Milk, yoghurt, custard, pudding, whipped cream, milk-based ice cream | Consume some dairy produce everyday | 0 grams a day or ≥ 750 grams a day | 300 - 450 grams a day |
| Fish | Lean fishes such as prawns and codfish, fatty fish such as salmon and anchovies | Consume one serving of fish every week, preferably oily fish | 0 grams a day | ≥ 15 grams a day |
| Tea | Green or black tea | Consume three cups of green or black tea everyday | 0 grams a day | ≥ 450 grams a day |
| Fats and oils | Butter, hard margarines, cooking fats, soft margarines, liquid cooking fats and plant oils | Replace butter, hard margarines, and cooking fats by soft margarines, liquid cooking fats, and vegetable oils | No consumption of soft margarines, liquid cooking fats and vegetable oils | No consumption of butter, hard margarines and cooking fats |
|  |  |  | OR | OR |
|  |  |  | Ratio of liquid cooking fats to solid cooking fats ≤ 0.6 | Ratio of liquid cooking fats to solid cooking fats ≥ 13 |
| Red meat | Beef, pork, lamb, horse, offal meats, hot dogs, hamburger and bacon | Restrict the consumption of red meat | ≥ 100 grams a day | ≤ 45 grams a day |
| Processed meat | Sausages and deli meats | Restrict the consumption of processed meat | ≥ 50 grams a day | 0 grams a day |
| Sweetened beverages and fruit juices | Carbonated sweetened beverages, noncarbonated sweetened beverages and fruit juices | Restrict the consumption of processed meat | ≥ 250 grams a day | 0 grams a day |
| Alcohol^b^ | Beer, wine, sherry, port, vermouth and strong alcoholic drinks | If alcohol is consumed, intake should be limited to one Dutch unit (i.e. 10 grams of ethanol) everyday | Women: ≥ 20 grams of ethanol a day  Men: ≥ 30 grams of ethanol a day | ≤ 10 grams of ethanol a day |

N/A; Not applicable

^a^ This component comprises two sub-components (1 and 2). Each sub-component has a maximum score of 5 points

^b^ This component was only added in the DHD15-index with alcohol component

Filtered or unfiltered coffee and salt intake was not assessed in the FFQ-NL 1.0

A continuous score with a minimum score of 0 and a maximum score of 10 is possible for each DHD15-index component. For example, a person with a mean intake of 55 grams of vegetables a day received a score of 2.75 for the vegetables component.

# Supplementary Table 2: DASH diet components with the corresponding foods for each component and the mean intake for the lowest and highest quintiles, adapted from Fung et al.

| **Components** | **Food items in DASH components** | **Quintile 1, mean grams per day** | **Quintile 5, mean grams per day** |
| --- | --- | --- | --- |
|  |  |  |  |
| Vegetables | All vegetables except for potatoes and legumes | 26.34 | 249.22 |
| Fruit | All fruits (not including fruit juices) | 20.37 | 344.27 |
| Wholegrains | Whole grain bread | 0.50 | 159.38 |
| Dairy | Low fat dairy products including skim milk and skim-milk cheese | 26.70 | 564.27 |
| Nuts and legumes | Nuts, seeds, seeds, peanut butter, beans and peas | 1.80 | 83.23 |
| Red and processed meats | Beef, pork, lamb, horse, organ meats, hot dogs, hamburger, bacon sausages and deli meats | 175.72 | 14.76 |
| Sweetened beverages | Carbonated and noncarbonated sweetened beverages | 201.93 | 0.00 |

For the DASH diet score, quintiles were calculated according to intake ranking. The component score for fruits, vegetables, nuts and legumes, low-fat dairy products, and whole grains ranged from 1 point (quintile 1) to 5 points (quintile 5) is assigned 1 point. Low intakes for red and processed meats and sweetened beverages were desired. Therefore, the lowest quintile was given a score of 5 points and the highest quintile, 1 point.

# Supplementary Table 3: Results regarding the mediating role of dietary cost in the association between individual and household educational level and the DHD15-index and DASH score

| **Educational level** | **Dietary cost** | **Diet quality** | **Educational level on dietary cost**  **(a-path)** | **Dietary cost on diet quality**  **(b-path)** | **Direct effect**  **(c’-path)** | | **Total effect**  **(c-path)** | | **Indirect effect**  **(a-path x b-path)** | | **Proportion mediated** |
| --- | --- | --- | --- | --- | --- | --- | --- | --- | --- | --- | --- |
|  |  |  |  |  | **β** | **95%CI** | **β** | **95%CI** | **β** | **Bootstrap 95%CI** | $\frac{\mathrm{AB}}{(C^{'}+AB)}$ |
|  | Dietary cost (€/d) | DHD15- index (score) | Individual educational level^a^ | | | | | | | | |
| Middle vs. low |  |  | 0.24* | 0.74* | 3.78* | 2.75; 4.82 | 3.96* | 2.93; 5.00 | 0.18* | 0.10; 0.28 | 4.5 |
| High vs. low |  |  | 0.28* |  | 8.86* | 8.16; 9.57 | 9.07* | 8.37; 9.77 | 0.20* | 0.11; 0.30 | 2.2 |
| High vs. middle |  |  | 0.03 |  | 5.07* | 4.00; 6.16 | 5.10* | 4.01; 6.19 | 0.02 | -0.03; 0.09 | N/A |
|  |  |  | Household educational level^b^ | | | | | | | | |
| Middle vs. low |  |  | 0.17* | 0.70* | 2.84* | 1.79; 3.89 | 2.96* | 1.91; 4.01 | 0.12* | 0.05; 0.21 | 4.1 |
| High vs. low |  |  | 0.30* |  | 8.04* | 7.37; 8.70 | 8.25* | 7.59; 8.90 | 0.21* | 0.11; 0.32 | 2.5 |
| High vs. middle |  |  | 0.12* |  | 5.20* | 4.15; 6.24 | 5.28* | 4.24; 6.33 | 0.09* | 0.03; 0.16 | 1.7 |
|  |  | DASH diet (score) | Individual educational level^a^ | | | | | | | | |
| Middle vs. low |  |  | 0.24* | 0.29* | 0.93* | 0.64; 1.22 | 1.00* | 0.71; 1.29 | 0.07* | 0.04; 0.10 | 7.0 |
| High vs. low |  |  | 0.28* |  | 2.17* | 1.98; 2.37 | 2.25* | 2.06; 2.45 | 0.08* | 0.05; 0.11 | 3.6 |
| High vs. middle |  |  | 0.03 |  | 1.24* | 0.94; 1.54 | 1.25* | 0.95; 1.56 | 0.01 | -0.01; 0.03 | N/A |
|  |  |  | Household educational level^b^ | | | | | | | | |
| Middle vs. low |  |  | 0.17* | 0.28* | 0.71* | 0.41; 1.00 | 0.75* | 0.46; 1.05 | 0.05* | 0.03; 0.08 | 6.6 |
| High vs. low |  |  | 0.30* |  | 1.94* | 1.76; 2.12 | 2.02* | 1.84; 2.21 | 0.08* | 0.05; 0.11 | 4.0 |
| High vs. middle |  |  | 0.12* |  | 1.23* | 0.94; 1.52 | 1.27* | 0.98; 1.56 | 0.03* | 0.01; 0.06 | 2.4 |

Abbreviations: B; beta regression coefficient, CI; confidence interval

^a^ Sample size for analyses with individual educational level is 9,275

^b^ Sample size for analyses with household educational level is 9,282

* *P*<0•05

# Supplementary Table 4: Results regarding the mediating role of dietary cost in the association between individual and household educational level and the DHD15-index and DASH score for participants ≤ 65 years of age.

| **Educational level** | **Dietary cost** | **Dietary quality** | **Educational level on dietary cost**  **(a-path)** | **Dietary cost on dietary quality**  **(b-path)** | **Direct effect**  **(c’-path)** | | **Total effect**  **(c-path)** | | **Indirect effect**  **(a-path x b-path)** | | **Proportion mediated** |
| --- | --- | --- | --- | --- | --- | --- | --- | --- | --- | --- | --- |
|  |  |  |  |  | **β** | **95%CI** | **β** | **95%CI** | **β** | **Bootstrap 95%CI** | $\frac{\boldsymbol{AB}}{\boldsymbol{(}\boldsymbol{C}^{\boldsymbol{'}}\boldsymbol{+AB)}}$ |
|  |  |  | Individual educational level^a^ | | | | | | | | |
| Middle vs. low | Dietary cost (€/d) | DHD15- index (score) | 0.18* | 0.37 | 5.07* | 3.36; 6.79 | 5.14* | 3.43; 6.85 | 0.07 | -0.03; 0.20 | N/A |
| High vs. low |  |  | 0.20* |  | 10.89* | 9.53; 12.26 | 10.97* | 9.60; 12.33 | 0.07 | -0.03; 0.20 | N/A |
| High vs. middle |  |  | 0.02 |  | 5.82* | 4.12; 7.53 | 5.83* | 4.12; 7.53 | 0.01 | -0.05; 0.07 | N/A |
|  |  |  | Household educational level^b^ | | | | | | | | |
| Middle vs. low |  |  | 0.14* | 0.27 | 4.27* | 2.47; 6.07 | 4.31* | 2.51; 6.10 | 0.04 | -0.04; 0.15 | N/A |
| High vs. low |  |  | 0.27* |  | 10.59* | 9.20; 11.98 | 10.66* | 9.28; 12.05 | 0.07 | -0.07; 0.22 | N/A |
| High vs. middle |  |  | 0.12 |  | 6.32* | 4.63; 8.00 | 6.35* | 4.67; 8.03 | 0.03 | -0.03; 0.13 | N/A |
|  |  |  | Individual educational level^a^ | | | | | | | | |
| Middle vs. low |  | DASH diet (score) | 0.18* | 0.20* | 1.21* | 0.73; 1.68 | 1.25* | 0.77; 1.72 | 0.04* | 0.01; 0.08 | 3.2 |
| High vs. low |  |  | 0.20* |  | 2.64* | 2.26; 3.02 | 2.68* | 2.30; 3.06 | 0.04* | 0.01; 0.08 | 1.5 |
| High vs. middle |  |  | 0.02 |  | 1.43* | 0.96; 1.91 | 1.43* | 0.96; 1.90 | 0.00 | -0.03; 0.03 | N/A |
|  |  |  | Household educational level^b^ | | | | | | | | |
| Middle vs. low |  |  | 0.14* | 0.17* | 1.01* | 0.51; 1.51 | 1.03* | 0.54; 1.53 | 0.02 | -0.00; 0.06 | N/A |
| High vs. low |  |  | 0.27* |  | 2.61* | 2.23; 3.00 | 2.66* | 2.27; 3.04 | 0.05* | 0.01; 0.09 | 1.9 |
| High vs. middle |  |  | 0.12 |  | 1.60* | 1.13; 2.07 | 1.62* | 1.16; 2.09 | 0.02 | -0.00; 0.06 | N/A |

Abbreviations: B; beta regression coefficient, CI; confidence interval, N/A; Not Applicable

^a^ Sample size for analyses with individual educational level is 2,413

^b^ Sample size for analyses with household educational level is 2,418

* *P*<0•05

All analyses were adjusted for sex, study center and energy intake

# Supplementary Table 5: Results regarding the mediating role of dietary cost in the association between individual and household educational level and the DHD15-index and DASH score for participants over 65 years of age.

| **Educational level** | **Dietary cost** | **Dietary quality** | **Educational level on dietary cost**  **(a-path)** | **Dietary cost on dietary quality**  **(b-path)** | **Direct effect**  **(c’-path)** | | **Total effect**  **(c-path)** | | **Indirect effect**  **(a-path x b-path)** | | **Proportion mediated** |
| --- | --- | --- | --- | --- | --- | --- | --- | --- | --- | --- | --- |
|  |  |  |  |  | **β** | **95%CI** | **β** | **95%CI** | **β** | **Bootstrap 95%CI** | $\frac{\boldsymbol{AB}}{\boldsymbol{(}\boldsymbol{C}^{\boldsymbol{'}}\boldsymbol{+AB)}}$ |
|  |  |  | Individual educational level^a^ | | | | | | | | |
| Middle vs. low | Dietary cost (€/d) | DHD15- index (score) | 0.29* | 0.88* | 3.37* | 2.07; 4.67 | 3.62* | 2.32; 4.92 | 0.25* | 0.13; 0.40 | 6.9 |
| High vs. low |  |  | 0.31* |  | 7.81* | 7.00; 8.63 | 8.08* | 7.27; 8.89 | 0.27* | 0.15; 0.41 | 3.3 |
| High vs. middle |  |  | 0.02 |  | 4.44* | 3.04; 5.83 | 4.46* | 3.06; 5.86 | 0.02 | -0.07; 0.11 | N/A |
|  |  |  | Household educational level^b^ | | | | | | | | |
| Middle vs. low |  |  | 0.21* | 0.86* | 2.68* | 1.38; 3.98 | 2.86* | 1.56; 4.16 | 0.18* | 0.08; 0.30 | 6.3 |
| High vs. low |  |  | 0.31* |  | 6.96* | 6.21; 7.71 | 7.23* | 6.48; 7.97 | 0.27* | 0.15; 0.40 | 3.7 |
| High vs. middle |  |  | 0.11* |  | 4.28* | 2.96; 5.61 | 4.37* | 3.05; 5.70 | 0.09* | 0.02; 0.19 | 2.1 |
|  |  |  | Individual educational level^a^ | | | | | | | | |
| Middle vs. low |  | DASH diet (score) | 0.29* | 0.32* | 0.76* | 0.40; 1.12 | 0.86* | 0.50; 1.22 | 0.09* | 0.06; 0.14 | 10.5 |
| High vs. low |  |  | 0.31* |  | 1.89* | 1.67; 2.12 | 1.99* | 1.77; 2.22 | 0.10* | 0.07; 0.14 | 5.0 |
| High vs. middle |  |  | 0.02 |  | 1.13* | 0.74; 1.51 | 1.13* | 0.75; 1.52 | 0.01 | -0.02; 0.04 | N/A |
|  |  |  | Household educational level^b^ | | | | | | | | |
| Middle vs. low |  |  | 0.21* | 0.32* | 0.63* | 0.27; 0.99 | 0.70* | 0.34; 1.06 | 0.07* | 0.03; 0.10 | 10.0 |
| High vs. low |  |  | 0.31* |  | 1.62* | 1.42; 1.83 | 1.72* | 1.52; 1.93 | 0.10* | 0.06; 0.14 | 5.8 |
| High vs. middle |  |  | 0.11* |  | 0.99* | 0.62; 1.36 | 1.03* | 0.66; 1.39 | 0.03* | 0.01; 0.07 | 2.9 |

Abbreviations: B; beta regression coefficient, CI; confidence interval

^a^ Sample size for analyses with individual educational level is 6,862

^b^ Sample size for analyses with household educational level is 6,864

* *P*<0•05

All analyses were adjusted for sex, study center and energy intake

# Supplementary Table 6: Results regarding the mediating role of dietary cost in the association between individual and household educational level and the DHD15-index and DASH score for females.

| **Educational level** | **Dietary cost** | **Dietary quality** | **Educational level on dietary cost**  **(a-path)** | **Dietary cost on dietary quality**  **(b-path)** | **Direct effect**  **(c’-path)** | | **Total effect**  **(c-path)** | | **Indirect effect**  **(a-path x b-path)** | | **Proportion mediated** |
| --- | --- | --- | --- | --- | --- | --- | --- | --- | --- | --- | --- |
|  |  |  |  |  | **β** | **95%CI** | **β** | **95%CI** | **β** | **Bootstrap 95%CI** | $\frac{\boldsymbol{AB}}{\boldsymbol{(}\boldsymbol{C}^{\boldsymbol{'}}\boldsymbol{+AB)}}$ |
|  |  |  | Individual educational level^a^ | | | | | | | | |
| Middle vs. low | Dietary cost (€/d) | DHD15- index (score) | 0.25* | 1.07* | 3.72* | 2.56; 4.88 | 3.99* | 2.83; 5.15 | 0.27* | 0.15; 0.40 | 6.8 |
| High vs. low |  |  | 0.26* |  | 8.17* | 7.37; 8.97 | 8.45* | 7.65; 9.25 | 0.28* | 0.18; 0.40 | 3.3 |
| High vs. middle |  |  | 0.01 |  | 4.45* | 3.22; 5.67 | 4.46* | 3.23; 5.69 | 0.01 | -0.07; 0.10 | N/A |
|  |  |  | Household educational level^b^ | | | | | | | | |
| Middle vs. low |  |  | 0.19* | 1.02* | 2.47* | 1.27; 3.68 | 2.67* | 1.46; 3.87 | 0.19* | 0.10; 0.31 | 7.1 |
| High vs. low |  |  | 0.29* |  | 7.28* | 6.54; 8.02 | 7.57* | 6.84; 8.30 | 0.29* | 0.18; 0.42 | 3.8 |
| High vs. middle |  |  | 0.10* |  | 4.80* | 3.61; 6.00 | 4.91* | 3.70; 6.10 | 0.10* | 0.02; 0.19 | 2.0 |
|  |  |  | Individual educational level^a^ | | | | | | | | |
| Middle vs. low |  | DASH diet (score) | 0.25* | 0.40* | 0.81* | 0.49; 1.14 | 0.91* | 0.59; 1.24 | 0.10* | 0.06; 0.14 | 11.0 |
| High vs. low |  |  | 0.26* |  | 1.93* | 1.71; 2.16 | 2.04* | 1.82; 2.26 | 0.11* | 0.07; 0.14 | 5.4 |
| High vs. middle |  |  | 0.01 |  | 1.12* | -0.78; 1.46 | 1.13* | 0.78; 1.47 | 0.01 | -0.03; 0.04 | N/A |
|  |  |  | Household educational level^b^ | | | | | | | | |
| Middle vs. low |  |  | 0.19* | 0.39* | 0.53* | 0.20; 0.87 | 0.61* | 0.27; 0.94 | 0.07* | 0.04; 0.11 | 11.7 |
| High vs. low |  |  | 0.29* |  | 1.67* | 1.47; 1.88 | 1.79* | 1.58; 1.99 | 0.11* | 0.08; 0.15 | 5.8 |
| High vs. middle |  |  | 0.10* |  | 1.14* | 0.81; 1.47 | 1.18* | 0.84; 1.51 | 0.04* | 0.01; 0.07 | 3.4 |

Abbreviations: B; beta regression coefficient, CI; confidence interval

^a^ Sample size for analyses with individual educational level is 7,175

^b^ Sample size for analyses with household educational level is 7,182

* *P*<0•05

All analyses were adjusted for age, study center and energy intake

# Supplementary Table 7: Results regarding the mediating role of dietary cost in the association between individual and household educational level and the DHD15-index and DASH score for males.

| **Educational level** | **Dietary cost** | **Dietary quality** | **Educational level on dietary cost**  **(a-path)** | **Dietary cost on dietary quality**  **(b-path)** | **Direct effect**  **(c’-path)** | | **Total effect**  **(c-path)** | | **Indirect effect**  **(a-path x b-path)** | | **Proportion mediated** |
| --- | --- | --- | --- | --- | --- | --- | --- | --- | --- | --- | --- |
|  |  |  |  |  | **β** | **95%CI** | **β** | **95%CI** | **β** | **Bootstrap 95%CI** | $\frac{\boldsymbol{AB}}{\boldsymbol{(}\boldsymbol{C}^{\boldsymbol{'}}\boldsymbol{+AB)}}$ |
|  |  |  | Individual educational level^a^ | | | | | | | | |
| Middle vs. low | Dietary cost (€/d) | DHD15- index (score) | 0.22* | -0.11 | 3.67* | 1.42; 5.92 | 3.65* | 1.40; 5.89 | -0.02 | -0.18; 0.10 | N/A |
| High vs. low |  |  | 0.29* |  | 10.31* | 8.85; 11.77 | 10.28* | 8.83; 11.73 | -0.03 | -0.21; 0.14 | N/A |
| High vs. middle |  |  | 0.07 |  | 6.64* | 4.36; 8.91 | 6.63* | 4.36; 8.91 | -0.01 | -0.08; 0.06 | N/A |
|  |  |  | Household educational level^b^ | | | | | | | | |
| Middle vs. low |  |  | 0.15 | -0.13 | 3.90* | 1.74; 6.06 | 3.88* | 1.72; 6.04 | -0.02 | -0.14; 0.08 | N/A |
| High vs. low |  |  | 0.31* |  | 9.88* | 8.40; 11.36 | 9.84* | 8.37; 11.31 | -0.04 | -0.24; 0.15 | N/A |
| High vs. middle |  |  | 0.16* |  | 6.00* | 3.86; 8.10 | 5.96* | 3.84; 8.08 | -0.02 | -0.14; 0.09 | N/A |
|  |  |  | Individual educational level^a^ | | | | | | | | |
| Middle vs. low |  | DASH diet (score) | 0.22* | 0.01 | 1.19* | 0.58; 1.81 | 1.20* | 0.59; 1.81 | 0.00 | -0.04; 0.04 | N/A |
| High vs. low |  |  | 0.29* |  | 2.72* | 2.32; 3.12 | 2.72* | 2.33; 3.12 | 0.00 | -0.05; 0.05 | N/A |
| High vs. middle |  |  | 0.07 |  | 1.52* | 0.90; 2.14 | 1.52* | 0.90; 2.14 | 0.00 | -0.02; 0.02 | N/A |
|  |  |  | Household educational level^b^ | | | | | | | | |
| Middle vs. low |  |  | 0.15 | 0.01 | 1.14* | 0.55; 1.73 | 1.14* | 0.55; 1.73 | 0.00 | -0.03; 0.03 | N/A |
| High vs. low |  |  | 0.31* |  | 2.59* | 2.18; 2.99 | 2.59* | 2.19; 2.99 | 0.00 | -0.05; 0.05 | N/A |
| High vs. middle |  |  | 0.16* |  | 1.45* | 0.87; 2.02 | 1.45* | 0.87; 2.03 | 0.00 | -0.03; 0.04 | N/A |

Abbreviations: B; beta regression coefficient, CI; confidence interval

^a^ Sample size for analyses with individual educational level is 2,100

^b^ Sample size for analyses with household educational level is 2,100

* *P*<0•05

All analyses were adjusted for age, study center and energy intake

# Supplementary Table 8: Results regarding the mediating role of dietary cost in the association between individual and household educational level and the DHD15-index and DASH score for participants older than 30 years at baseline

| **Educational level** | **Dietary cost** | **Dietary quality** | **Educational level on dietary cost**  **(a-path)** | **Dietary cost on dietary quality**  **(b-path)** | **Direct effect**  **(c’-path)** | | **Total effect**  **(c-path)** | | **Indirect effect**  **(a-path x**  **b-path)** | | **Proportion mediated** |
| --- | --- | --- | --- | --- | --- | --- | --- | --- | --- | --- | --- |
|  |  |  |  |  | **β** | **95%CI** | **β** | **95%CI** | **β** | **Bootstrap 95%CI** | $\frac{\boldsymbol{AB}}{\boldsymbol{(}\boldsymbol{C}^{\boldsymbol{'}}\boldsymbol{+AB)}}$ |
|  | Dietary cost (€/d) |  | Individual educational level^a^ | | | | | | | | |
| Middle vs. low |  | DHD15- index | 0.29* | 0.62* | 3.52* | 2.40; 4.63 | 3.70* | 2.59; 4.81 | 0.18* | 0.08; 0.30 | 4.9 |
| High vs. low |  |  | 0.27* |  | 8.71* | 8.00; 9.43 | 8.87* | 8.16; 9.59 | 0.16* | 0.08; 0.26 | 1.8 |
| High vs. middle |  |  | -0.02 |  | 5.19* | 4.01; 6.37 | 5.18* | 4.00; 6.35 | -0.01 | -0.07; 0.04 | N/A |
|  |  |  | Household educational level^b^ | | | | | | | | |
| Middle vs. low |  |  | 0.21* | 0.58* | 2.61* | 1.49; 3.74 | 2.73* | 1.61; 3.86 | 0.12* | 0.05; 0.21 | 4.4 |
| High vs. low |  |  | 0.29* |  | 7.88* | 7.20; 8.56 | 8.05* | 7.37; 8.72 | 0.17* | 0.07; 0.27 | 2.1 |
| High vs. middle |  |  | 0.08* |  | 5.27* | 4.13; 6.40 | 5.31* | 4.18; 6.45 | 0.05* | 0.00; 0.11 | 0.9 |
|  |  |  | Individual educational level^a^ | | | | | | | | |
| Middle vs. low |  | DASH diet | 0.29* | 0.25* | 0.86* | 0.55; 1.17 | 0.93* | 0.63; 1.24 | 0.07* | 0.04; 0.11 | 7.5 |
| High vs. low |  |  | 0.27* |  | 2.15* | 1.95; 2.35 | 2.21* | 2.02; 2.41 | 0.07* | 0.04; 0.10 | 3.2 |
| High vs. middle |  |  | -0.02 |  | 1.29* | 0.97; 1.62 | 1.29* | 0.96; 1.61 | -0.01 | -0.03; 0.01 | N/A |
|  |  |  | Household educational level^b^ | | | | | | | | |
| Middle vs. low |  |  | 0.21* | 0.24* | 0.66* | 0.35; 0.98 | 0.71* | 0.40; 1.03 | 0.05* | 0.03; 0.08 | 7.0 |
| High vs. low |  |  | 0.29* |  | 1.91* | 1.72; 2.10 | 1.98* | 1.79; 2.17 | 0.07* | 0.04; 0.10 | 3.5 |
| High vs. middle |  |  | 0.08* |  | 1.25* | 0.93; 1.56 | 1.27* | 0.95; 1.58 | 0.02* | 0.00; 0.04 | 1.6 |

Abbreviations: B; beta regression coefficient, CI; confidence interval

^a^ Sample size for analyses with individual educational level is 8,746

^b^ Sample size for analyses with household educational level is 8,751

* *P*<0•05

All analyses were adjusted for age, study center and energy intake

# Supplementary Table 9: Results regarding the mediating role of dietary cost in the association between individual and household educational level and the DHD15-index with alcohol component

| **Educational level** | **Dietary cost** | **Dietary quality** | **Educational level on dietary cost**  **(a-path)** | **Dietary cost on dietary quality**  **(b-path)** | **Direct effect**  **(c’-path)** | | **Total effect**  **(c-path)** | | **Indirect effect**  **(a-path x b-path)** | | **Proportion mediated** |
| --- | --- | --- | --- | --- | --- | --- | --- | --- | --- | --- | --- |
|  |  |  |  |  | **β** | **95%CI** | **β** | **95%CI** | **β** | **Bootstrap 95%CI** | $\frac{\boldsymbol{AB}}{\boldsymbol{(}\boldsymbol{C}^{\boldsymbol{'}}\boldsymbol{+AB)}}$ |
|  |  |  | Individual educational level^a^ | | | | | | | | |
| Middle vs. low | Dietary cost (€/d) | DHD15- index with alcohol | 0.24* | -0.78* | 2.14* | 0.99; 3.29 | 1.95* | 0.80; 3.10 | -0.19* | -0.30; -0.10 | N/A |
| High vs. low |  |  | 0.28* |  | 7.35* | 6.57; 8.13 | 7.14* | 6.37; 7.91 | -0.21* | -0.32; -0.12 | N/A |
| High vs. middle |  |  | 0.03 |  | 5.22* | 4.01; 6.42 | 5.19* | 4.00; 6.39 | -0.03 | -0.09; 0.03 | N/A |
|  |  |  | Household educational level^b^ | | | | | | | | |
| Middle vs. low |  |  | 0.17* | -0.83* | 1.92* | 0.75; 3.08 | 1.77* | 0.61; 2.94 | -0.14* | -0.24; -0.08 | N/A |
| High vs. low |  |  | 0.30* |  | 6.65* | 5.91; 7.38 | 6.40* | 5.68; 7.13 | -0.25* | -0.36; -0.14 | N/A |
| High vs. middle |  |  | 0.12* |  | 4.73* | 3.58; 5.89 | 4.63* | 3.47; 5.79 | -0.10* | -0.19; -0.04 | N/A |

Abbreviations: B; beta regression coefficient, CI; confidence interval

^a^ Sample size for analyses with individual educational level is 9,275

^b^ Sample size for analyses with household educational level is 9,282

* *P*<0•05

All analyses were adjusted for age, study center and energy intake
